# MicroRNA-99a Suppresses Breast Cancer Progression by Targeting FGFR3

**DOI:** 10.3389/fonc.2019.01473

**Published:** 2020-01-24

**Authors:** Xinghua Long, Yu Shi, Peng Ye, Juan Guo, Qian Zhou, Yueting Tang

**Affiliations:** Department of Laboratory Medicine, Zhongnan Hospital of Wuhan University, Wuhan, China

**Keywords:** miR-99a, FGFR3, breast cancer, metastasis, deep sequencing, lncRNA, circRNA

## Abstract

MicroRNAs have been implicated in acting as oncogenes or anti-oncogenes in breast cancer by regulating diverse cellular pathways. In the present study, we investigated the effects of miR-99a on cell biological processes in breast cancer. Breast cancer cells were transfected with a lentivirus that expressed miR-99a or a scramble control sequence. Functional experiments showed that miR-99a reduced breast cancer cell proliferation, invasion and migration. Tumor xenograft experiment suggested miR-99a overexpression inhibited breast cancer cell proliferation *in vivo*. The dual luciferase assay revealed that miR-99a directly targets FGFR3 by binding its 3′ UTR in breast cancer. miR-99a was strongly down-regulated in breast tumor and FGFR3 was significantly up-regulated in breast tumor. FGFR3 silencing inhibited proliferation, migration and invasion of breast cancer cells. Deep sequencing indicated that miR-99a overexpression regulates multiple signaling pathways and triggers the alteration of the whole transcriptome. We constructed correlated expression networks based on circRNA/miRNA and lncRNA/miRNA competing endogenous RNAs regulation and miRNA-mRNA interaction, which provided new insights into the regulatory mechanism of miR-99a. In conclusion, these results suggest that the miR-99a/FGFR3 axis is an important tumor regulator in breast cancer and might have potential as a therapeutic target.

## Introduction

MicroRNAs (miRNAs) are small non-coding RNA molecules containing 18–22 nucleotides that act as agents of the RNA interference pathway and can lead to silencing of their cognate target genes by targeting mRNAs for cleavage or translational repression (1). Gene regulation by miRNAs has been studied for over 15 years and has been extensively reviewed (2). Indeed, miRNAs play prominent regulatory roles in the output of 30–50% of protein-coding genes and often target various components of cellular networks or signaling pathways (3), and they are involved in the regulation of a variety of biological processes, including metabolism (4), apoptosis (5), and the cell cycle (6). Dysregulation of miRNAs is implicated in many human diseases including cancer.

Emerging evidence indicates that some miRNAs function as oncogenes or anti-oncogenes, which has led to a new strategy for cancer diagnostics and therapeutics. The miRNA expression profiles are very different between normal and tumor tissues, and miRNA signatures are associated with specific clinicobiological features (7). Breast cancer is one of the most common malignant tumors among women worldwide with high morbidity and mortality (8). MiRNA expression profile analyses have been used to reveal characteristic miRNA signatures in human breast cancers. The method of miRNA microarray has been used to show that miR-99a is down-regulated in breast tumor tissues compared with adjacent normal tissues (9). Furthermore, miR-99a has been described as participating in the pathogenesis of different malignancies, including leukemia (10), bladder cancer (11), prostate carcinoma (12), and non-small cell lung cancer (13). A small number of studies have confirmed the role of miR-99a in human breast cancer and have identified HOXA1 (14), mTOR (15), and IGFBP-1 (16) as its target genes. However, there is still much unknown about the role of miR-99a in breast cancer that needs to be studied.

In the present study, we explored the effects of miR-99a on cellular biological processes and identified FGFR3 as one of its target genes. Moreover, we elucidated the alteration of the whole transcriptome triggered by miR-99a using next-generation sequencing technology. Our study demonstrated that miR-99a could be a potential miRNA biomarker for breast cancer.

## Materials and Methods

### Cell Culture

The human breast cancer MCF-7 and MDA-MB-231 cell lines were obtained from the American Type Culture Collection (ATCC), and grown in Dulbecco's Modified Eagle Medium (DMEM) (HyClone, USA) supplemented with 10% fetal bovine serum (Gibco, USA) and 100 units/ml penicillin/streptomycin (Gibco, USA) at 37°C in a humidified atmosphere containing 5% CO_2_.

### Lentiviral Vector Construction and Transfection

For lentiviral construction, the hsa-miR-99a precursor sequence was cloned into the GV369 vector which contains a puromycin resistance gene. pHelper 1.0 and pHelper 2.0 are lentiviral packaging plasmids. The 293T cells were co-transfected with the recombinant GV369 plasmid, pHelper 1.0 and pHelper 2.0, and the medium was collected for the purification of viruses after 48 h. Breast cancer cell transfections were carried out at an MOI (multiplicity of infection) of 20 in the presence of 10 μg/ml polybrene. The culture medium was changed 12 h post-transfection and quantitative real-time polymerase chain reaction (qRT-PCR) was performed to determine miR-99a expression after another 72 h. The stably transfected cells were selected for 2 weeks by 5 μg/ml puromycin.

### Proliferation and Viability Assays

For the Cell Counting Kit-8 (CCK8) (Dojindo, Japan) assay, cells were seeded in 96-well-plates (Costar, NC) in quintuplicate at 1 × 10^3^ cells per well-containing 100 μl of Dulbecco's Modified Eagle Medium (HyClone, USA) supplemented with 10% fetal bovine serum (Gibco, USA). At 0, 24, 48, 72, 96, and 120 h, cell viability was measured with the CCK8 reagent according to the manufacturer's protocol. The colony formation assay was used to investigate the colony formation ability of cells. The cells (200 cells/dishes) were plated in 60 mm culture dishes and incubated for 14 days. The colonies were counted after being fixed with 100% methanol for 15 min and stained in a 0.1% crystal violet (Biosharp, USA) solution for 30 min.

### Migration and Invasion Assays

For the Transwell assay, 3 × 10^4^ cells were plated on the 24-well-top chambers (8 um-pore, Costar) either coated with 10 μg of Matrigel per well (for invasion assays) or uncoated (for *in vitro* migration assays) containing 100 μl of DMEM, and the lower chambers were filled with 600 μl of DMEM with 10% fetal bovine serum as a chemoattractant. After 24 h, the cells that did not migrate or invade through the pores were removed with a cotton swab, and the cells attached to the lower surface of the membrane were fixed with 100% methanol for 15 min and stained with 0.1% crystal violet (Biosharp) solution for 30 min. For the wound healing assay, 5 x 10^5^ cells were plated on a 6-well-plate in DMEM with 10% fetal bovine serum. After 24 h, wounds were created with a white micropipette tip and the medium was changed to DMEM with 2% fetal bovine serum. Light microscope pictures were taken at 10x magnification at 0, 12, 24, and 48 h. Wound distances were measured in three different places.

### Plasmid Construction and Luciferase Assay

For target validation, the 3′ UTR of FGFR3 was PCR-amplified and cloned downstream of firefly luciferase in the pmirGLO dual-luciferase miRNA target expression plasmid. The recombinant plasmid was sequenced to verify integrity. Three point mutations were obtained by using the QuikChange II XL Site-Directed Mutagenesis Kit (Stratagene, USA) according to the manufacturer's instructions. The 293T cells were co-transfected with the recombinant plasmids and miR-99a mimic or a scramble control. After 48 h, luciferase assays were performed according to the manufacturer's instructions (Promega, USA).

### RNA Isolation, RNA-Reverse Transcription, and qRT-PCR Analysis

Total RNA was isolated from the cultured cells using the Trizol reagent (Invitrogen, CA, USA) following the manufacturer's instructions. RNA was reverse transcribed into cDNA using oligo dT primers for mRNA or specific stem-loop reverse transcription primers for miRNA with a RevertAid First Strand cDNA Synthesis Kit (THERMO, USA). Then qRT-PCR was performed using the SYBR Green PCR Super Mix (BIO-RAD, USA) and the BIO-RAD CFX96 Real-Time PCR Detection System (BIO-RAD, USA). The expression levels of mRNAs were normalized to GAPDH as the internal control, while miRNAs were normalized to U6 snRNA, and expression levels were calculated using the 2-ΔΔCT algorithm.

### High Throughput Sequencing and Bioinformatics Analyses

Firstly, a Bioanalyzer 2100 (Agilent, USA) was used to check the quantity and purity of the total RNA. Then, microRNA libraries were generated using TruSeq Small RNA Sample Prep Kits (Illumina, USA). mRNA, lncRNA, and circRNA libraries were created according to the protocol of the mRNA-Seq Sample Preparation Kit (Illumina, USA), after removing the ribosomal RNA using the Epicentre Ribo-Zero Gold Kit (Illumina, USA). Finally, single-end sequencing for miRNA and paired-end sequencing for mRNA, lncRNA and circRNA were performed on the Illumina HiSeq 2500 platform and the Illumina Hiseq 4000 platform, respectively.

Data filtering was conducted on the raw reads output from the sequencing platform using Cutadapt (17) and clean reads were obtained. Bowtie2 (18) and Tophat2 (19) were used to map the reads to the genome of Homo sapiens, and String Tie (20) was used to estimate the expression levels of all transcripts. The original sequence counts were normalized to FPKM (fragments per kilo-base of exon per million fragments mapped). Differential expression analysis was performed using the EBseq (2010) R package, and |log_2_(Fold-Change)| ≥ 1 and *p* ≤ 0.05 as the threshold for significantly differential expression.

### Tumor Xenograft

Five-week-old female BALB/c nude mice were purchased from Huafukang (Beijing, China). All animal procedures in this study were approved by the Animal Ethics Committee of Wuhan Myhalic Biotechnology Co., Ltd., and performed based on the “Guidelines for Animal Care and Use of the Model Animal Research Institute.” MCF-7 cells transfected with miR-99a or the scramble control sequence were resuspended in PBS and injected into each mouse *in situ*, respectively (1 × 10^6^ cells/0.1 ml PBS per mouse, 5 mice in each group). Tumor volumes were assessed by caliper measurements and calculated as: V = D × d^2^ × 0.5 (D, the longer diameter; d, the shorter diameter), each formed tumor was measured every 5 days with calipers. The mice were euthanized 35 days after the injection, and the tumors were weighed.

### Statistical Analysis

Statistical analysis was performed with GraphPad Prism 5.0. Two tailed unpaired *t*-tests were employed, and the data are represented as means ± SEM. *p* < 0.05 were statistically significant (^*^p < 0.05 and ^**^p < 0.01). All the experiments were executed at least in triplicate and in independent biological replicates.

## Results

### MiR-99a and Its Target FGFR3 Expression Profile in Breast Cancer

**Figure 1:**
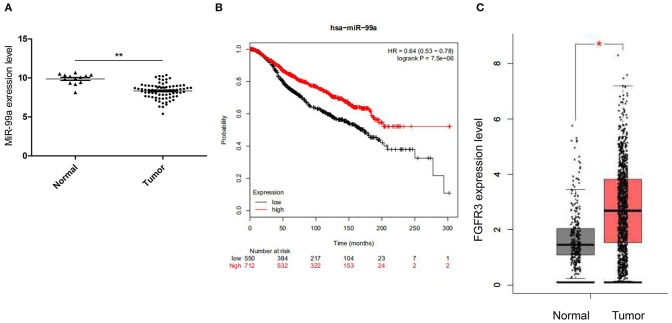
**(A)** Relative miR-99a expression in breast cancer tissues retrieved from GEO database. **(B)** The overall survival rates of breast cancer patients with low or high expression levels of miR-99a were estimated with the Kaplan–Meier method by log-rank test according to data from the Cancer Genome Atlas (TCGA). **(C)** Relative FGFR3 expression in breast cancer tissues retrieved from TCGA and the GTEx (*P < 0.05 and **P < 0.01).

The Gene Expression Omnibus database (GEO, http://www.ncbi.nlm.nih.gov/geo/) of the National Center for Biotechnology Information (NCBI) is an international public repository for microarray and next-generation sequencing functional genomic data sets submitted by the research community. We retrieved the microarray data set GSE26659 (21) containing 77 ductal breast carcinoma biopsies and 17 mammoplasties from the GEO database and identified the differentially expressed miRNAs. Results from this data set showed that miR-99a was strongly down-regulated in breast tumor tissues compared with the adjacent normal tissues (Figure 1A). The KM plotter (http://www.kmplot.com) was used to evaluate survival analysis between miR-99a expression level and breast cancer (22), we found that lower expression of miR-99a is related to shorter overall survival in breast cancer (Figure 1B). FGFR3 is a target of miR-99a, FGFR3 expression profile was analyzed by GEPIA, GEPIA (Gene Expression Profiling Interactive Analysis) is an interactive web application that analyzes the RNA sequencing expression data of more than 9,000 tumors and 8,000 normal samples from The Cancer Genome Atlas (TCGA) and the GTEx projects (http://gepia.cancer-pku.cn/) (23). We retrieved the data containing 1,085 breast tumor and 291 normal control from GEPIA, it showed that FGFR3 was significantly up-regulated in breast tumor (Figure 1C). These results suggested that miR-99a expression was inversely associated with its target FGFR3 in breast cancer.

### MiR-99a Negatively Regulates Breast Cancer Cell Proliferation

**Figure 2:**
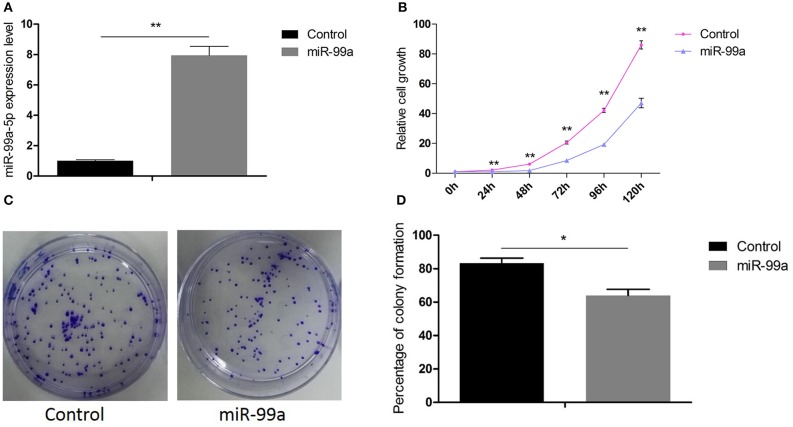
Overexpression of miR-99a inhibited proliferation of MCF-7 cells. **(A)** qRT-PCR of miR-99a in MCF-7 cells transfected with miR-99a or the scramble control sequence. **(B)** Cell viability was determined by CCK8 assay in MCF-7 cells. **(C,D)** Cell viability was determined by colony formation assay in MCF-7 cells. Means of three independent experiments ± SEM were shown (*P < 0.05 and **P < 0.01).

Firstly, we performed qRT-PCR to confirm the transfection efficiency of lentiviral-miR-99a and lentiviral control. The results indicated that the level of mature miR-99a was increased by 7.95-fold in MCF-7 cells transfected by lentiviral-miR-99a compared to control cells (Figure 2A). The role of miR-99a in MCF-7 cell proliferation was assessed by CCK8 and colony formation assays. The CCK8 assay indicated that miR-99a over-expression significantly reduced cell viability (Figure 2B). The colony formation assay showed significantly fewer colonies in cells over-expressing miR-99a than in control cells (Figures 2C,D). The same experiments were repeated and confirmed in MDA-MB-231 breast cancer cell line, the results were shown in Figure S1.

### MiR-99a Overexpression Reduces Breast Cancer Cell Migration and Invasion

**Figure 3:**
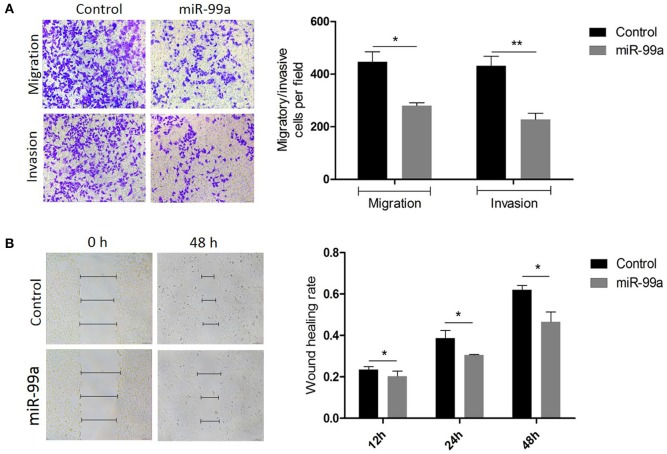
Overexpression of miR-99a inhibited migration and invasion of MCF-7 cells. **(A)** Transwell migration and matrigel invasion assays on MCF-7 cells transfected with miR-99a or scramble-control. **(B)** Wound healing assay on MCF-7 cells in which miR-99a over-expression or not. Wounds detected in 0, 12 and 48 h were represented as a graph. Means of three independent experiments ± SEM were shown (*P < 0.05 and **P < 0.01).

The ability of miR-99a to regulate migration and invasion was investigated using the Transwell assay. We observed that cells over-expressing miR-99a showed significantly reduced migration and invasion rates compared with the control-miR transfected cells (Figure 3A). We also used a wound healing assay to investigate the function of miR-99a in cell biological processes. As shown in Figure 3B, the migration distance of cells over-expressing miR-99a was significantly shorter than that of the control-miR transfected cells. In all experiments, the over-expression of miR-99a markedly decreased both the migration and the invasion of MCF-7 cells *in vitro*. The same experiments were repeated and confirmed in MDA-MB-231 breast cancer cell line, the results were shown in Figure S2.

### MiR-99a Directly Targets FGFR3 in Breast Cancer

**Figure 4:**
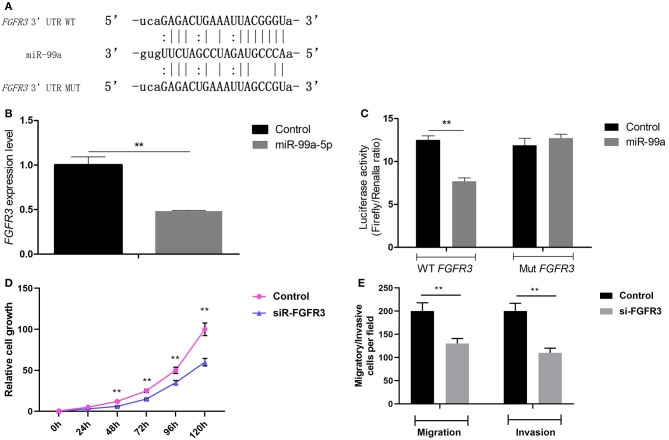
FGFR3 is a direct target gene of miR-99a. **(A)** The putative binding site of miR-99a in the FGFR3 3′ UTR, and the mutated 3′ UTR. **(B)** qRT-PCR of FGFR3 in MCF-7 cells transfected with miR-99a or the scramble control sequence. **(C)** The luciferase activity of 293T cells was detected after the pmirGLO plasmids with WT or Mut FGFR3 3′ UTR genes were co-transfected with miR-NC or miR-99a mimics. **(D)** FGFR3 silencing inhibited proliferation of MCF-7 cells. MCF-7 cells were transfected FGFR3 siRNA (siR-FGFR3) or the scramble control siRNA. Cell viability was determined by CCK8 assay in MCF-7 cells. **(E)** FGFR3 silencing inhibited migration and invasion of MCF-7 cells. Transwell migration and matrigel invasion assays on MCF-7 cells were transfected FGFR3 siRNA or the scramble control siRNA. Means of three independent experiments ± SEM were shown (**P < 0.01).

To understand the mechanisms by which miR-99a induces tumor invasion and metastasis, TargetScan, MiRanda, and PicTar were employed to identify putative targets of miR-99a-5p. FGFR3 was predicted to be a target gene of miR-99a by these three search programs (Figure 4A). To assess the validity of the prediction, we performed qRT-PCR to evaluate differences in FGFR3 mRNA expression between MCF-7 cells transfected with miR-99a and those transfected with a scramble control. We found that the mRNA expression of FGFR3 was higher in the cells with a low expression level of miR-99a (Figure 4B). To investigate whether miR-99a directly interacts with the 3′ UTR of FGFR3, a luciferase assay was performed. We found that the normalized activity of a firefly luciferase reporter gene with the wild-type FGFR3 3′ UTR inserted downstream was significantly reduced by the expression of miR-99a; however, the activity of a luciferase reporter that carried a mutant FGFR3 3′ UTR (with substitution of three nucleotides within the miR-99a binding site) showed no significant change (Figure 4C). This demonstrates that there is specific binding of miR-99a to the 3′ UTR of FGFR3. Small interfering RNA-mediated FGFR3 knockdown resulted in inhibition of MCF-7 cells growth and markedly decreased both the migration and the invasion of MCF-7 cells *in vitro* (Figures 4D,E). The small interfering RNA-mediated FGFR3 knockdown experiments were repeated and confirmed in MDA-MB-231 breast cancer cell line, the results were shown in Figure S3.

### MiR-99a Overexpression Regulates Multiple Signaling Pathways and Triggers Alteration of the Entire Transcriptome

**Figure 5:**
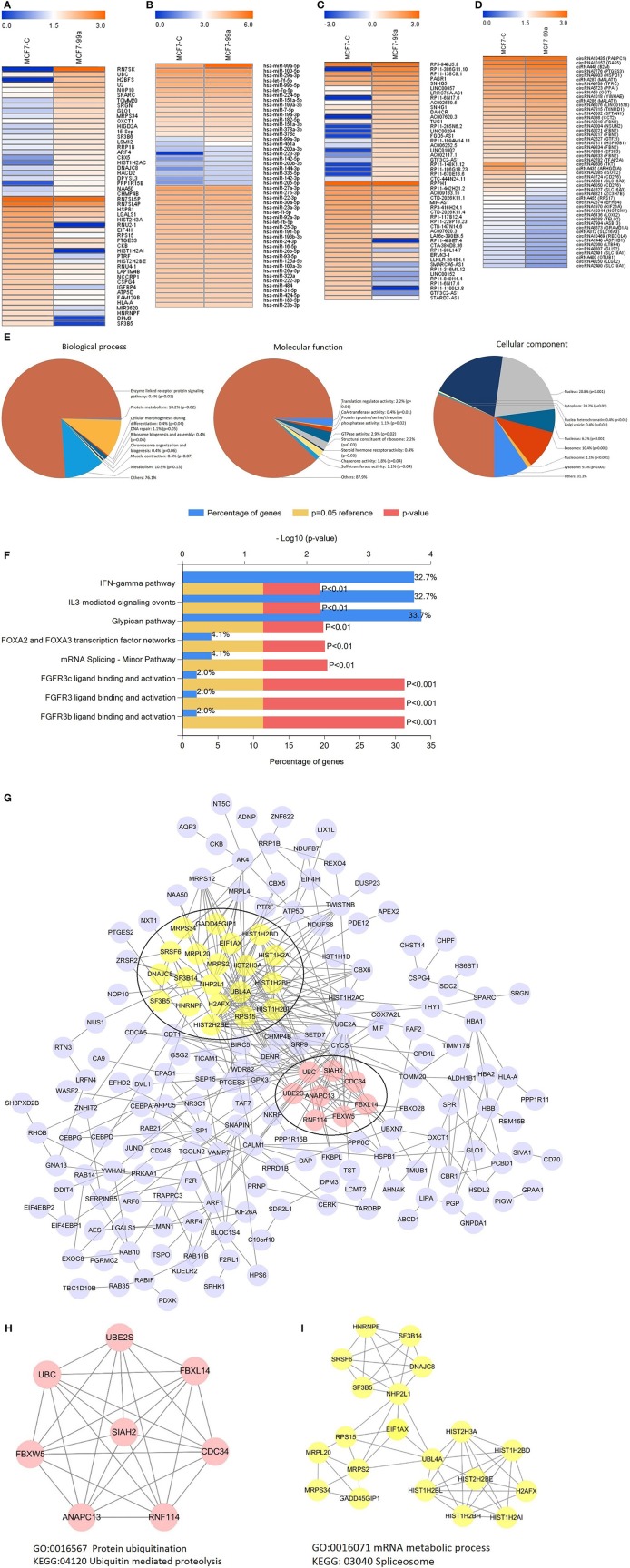
Heatmaps showing the expression profiles of mRNAs **(A)**, miRNAs **(B)**, lncRNAs **(C)**, and circRNAs **(D)**. The top25 expression levels of up-regulated RNAs and the top25 expression levels of down-regulated RNAs were listed. The color bars refer to the log10FPKM of RNAs. Gene Ontology (GO) analysis of differentially expressed mRNAs **(E)**. Biological pathway analysis of differentially expressed mRNAs **(F)**. Differential expression mRNAs protein-protein interaction network **(G)** and modular analysis **(H,I)**. MCF7-C, MCF-7 cells transfected with scramble-control sequence; MCF7-99a, MCF-7 cells transfected with miR-99a.

The effects of genes are not single, but widespread. Therefore, we performed next-generation sequencing of the transcriptome to investigate the underlying mechanism of the effect of miR-99a in breast cancer. The results revealed that miR-99a overexpression results in the dysregulation of a series of mRNAs, miRNAs, lncRNAs and circRNAs. The heatmaps (Figures 5A–D) provided a visual representation of the numbers of genes with the most obvious differences between cells overexpressing miR-99a and control cells.

To better understand the principal functions of the differentially expressed mRNAs, we performed Gene Ontology (GO) and biological pathway enrichment analyses using FunRich (Functional Enrichment Analysis Tool) (24) version 2.1.2. The GO analysis showed that these mRNAs were primarily enriched in the biological processes including enzyme linked receptor protein signaling pathway, protein metabolism, cellular morphogenesis during differentiation, etc.; cellular components including nucleus, cytoplasm, extracellular exosome, etc.; and molecular functions including translation regulator activity, CoA-transferase activity, GTPase activity, etc. (Figure 5E). Additionally, these differentially expressed mRNAs were enriched in FGFR3 ligand binding and activation, IFN-γ pathway, glypican pathway, etc. (Figure 5F). We also executed a PPI (protein-protein interaction network) analysis of all differentially expressed mRNAs to identify the hub genes by using the STRING online database. Among the PPI network, 28 central node genes of 2 modules were identified by filtering of intensive interactions, including CDC34, ANAPC13, NHP2L1, and so on (Figure 5G). GO and biological pathway enrichment analyses showed that Module 1 consisted of 8 nodes and 20 edges (Figure 5H), which were mainly associated with protein ubiquitination and ubiquitin mediated proteolysis, and that Module 2 consisted of 20 nodes and 57 edges (Figure 5I), which were mainly associated with mRNA metabolic process and spliceosome.

**Figure 6:**
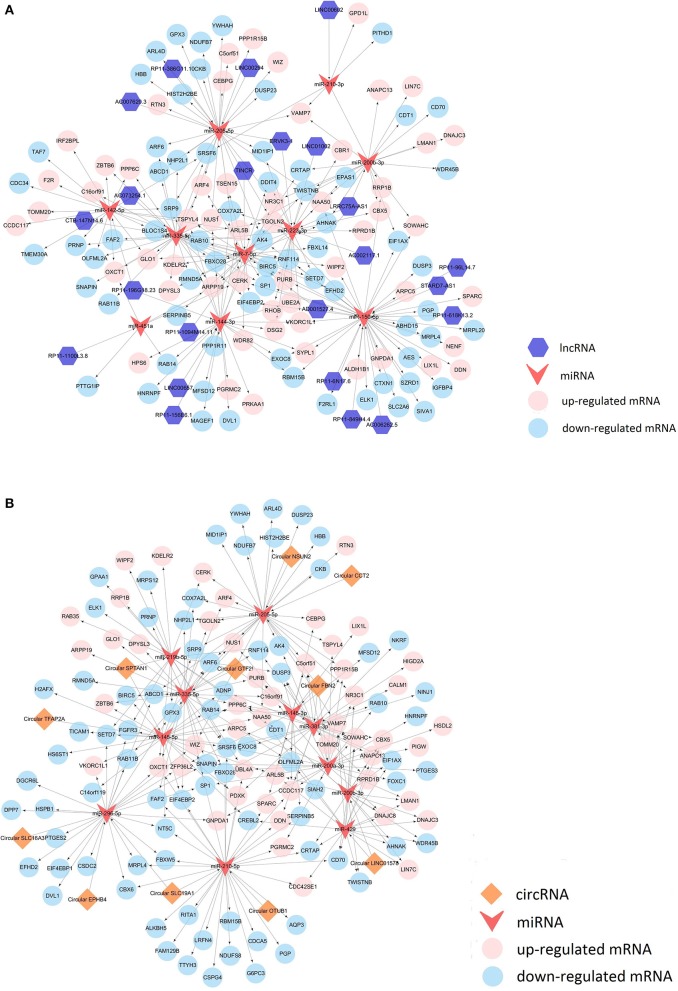
LncRNA competing endogenous RNA network **(A)** and circRNA competing endogenous RNA network **(B)**.

In addition to mRNA, there were also alterations in the expression of other RNAs between the MCF-7 cells overexpressing miR-99a and the scramble-control MCF-7 cells. To connect these differentially expressed transcriptome RNAs, we constructed correlated expression networks based on the relationships of circRNA/miRNA and lncRNA/miRNA competing endogenous RNAs regulation and miRNA-mRNA interactions (Figure 6).

### MiR-99a Inhibits Breast Cancer Proliferation *in vivo*

**Figure 7:**
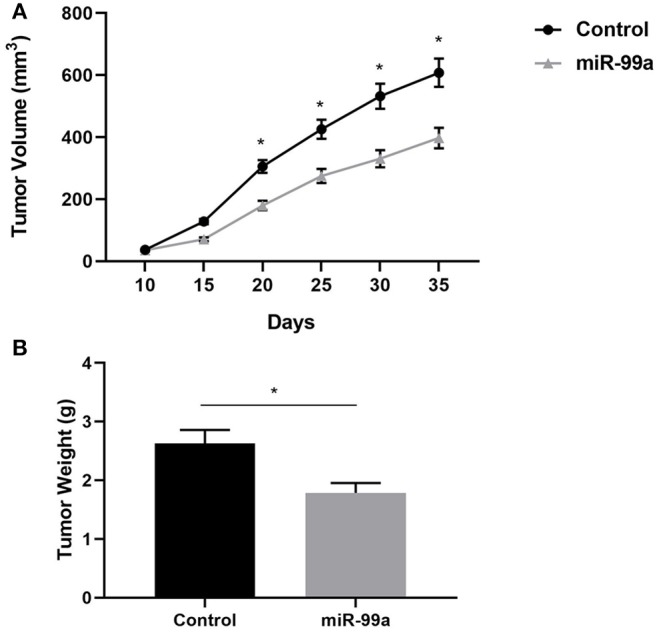
miR-99a inhibits breast cancer proliferation *in vivo*. **(A)** Growth kinetics of tumors in nude mice. Tumor diameters were measured every 5 days. **(B)** Average weight of tumors in nude mice. Mean ± SEM were shown (*P < 0.05).

To determine the role of miR-99a in breast cancer progression *in vivo*, BALB/c nude mice were injected with MCF-7 cells transfected with miR-99a or the scramble control sequence. We found that miR-99a overexpression significantly inhibited tumor growth *in vivo*, the tumor volumes and weights of the miR-99a overexpressed group were significantly lower than that of the control group (Figure 7).

## Discussion

MiR-99a is located in intron 13 of the C21orf34 gene on chromosome 21q (25). In the present study, we found that miR-99a is downregulated in primary breast tumors relative to normal breast tissues by analyzing miRNA expression profiles from the GEO database. This result was supported by data from the OncomiR database (26). Interestingly, in addition to breast cancer, down-regulation of miR-99a was also frequently seen in other types of cancer such as bladder urothelial carcinoma, cholangiocarcinoma, esophageal carcinoma, and head and neck squamous cell carcinoma, suggesting the potential involvement of miR-99a in the tumorigenesis of many kinds of cancers.

By over-expressing miR-99a in breast cancer cells, we confirmed that miR-99a inhibited the proliferative, invasive and migratory activities of breast cancer cells. Analysis based on TCGA database also suggests that low expression of mir-99a predicts a poor outcome in breast cancer. Our results were supported by those of other several studies in which miR-99a was shown to reduce the aggressiveness of breast cancer (15, 16, 27). This implies that miR-99a acts as a tumor suppressor in breast cancer. Moreover, it has been reported that low levels of serum miR-99a were associated with clinical outcomes and could serve as a predictor of poor prognosis in breast cancer (28).

Previous studies revealed that mTOR, HOXA1, and IGFBP1 were targeted by miR-99a in breast cancer (14–16); FGFR3 was targeted by miR-99a in bladder cancer (29); in the present study, we identified FGFR3 as a direct target of miR-99a in breast cancer for the first time. The fibroblast growth factor receptor 3 (FGFR3) gene, which is located on chromosome 4p16.3, encodes a protein whose extracellular portion can interact with fibroblast growth factors and initiate a cascade of downstream signals that regulates mitogenesis and differentiation (28, 30). Overexpression of FGFR3 is frequently observed in various cancers, and abnormal expression of FGFR3 could directly or indirectly activate various downstream signaling pathways, such as the FGFR3 signaling pathway (31), the PI3K-AKT signaling pathway (32), and the RAS/RAF/MEK/MAPK pathway (33), which are key mediators of the occurrence and development of malignant tumors. FGFR3 was significantly up-regulated in breast tumor according to data retrieved from TCGA and the GTEx. Furthermore, the activation of FGFR3 reduced the sensitivity of breast cancer cells to tamoxifen and fulvestrant (34) and might serve as a candidate therapeutic target gene (35). In this paper, the relationship between the expression of miR-99a and that of FGFR3 was negatively correlated in breast cancer, and FGFR3 was directly regulated by miR-99a. We speculate that the down-regulation of miR-99a is implicated in breast cancer carcinogenesis and progression through the regulation of FGFR3.

Despite some functional experiments and the identification of target genes, the biological mechanism of miR-99a remains poorly understood. To elucidate the underlying molecular mechanisms of miR-99a in breast cancer, we applied the deep sequencing method to detect transcriptome changes triggered by miR-99a overexpression in the MCF-7 cell line. In brief, the results demonstrated that miR-99a overexpression initiated wide-ranging alterations of the transcriptome in MCF-7 cells. Among the down-regulated mRNAs, some genes with potential oncogenic functions could be found, such as FGFR3 and EXOC8, which are also putative target genes of miR-99a. As previously mentioned, FGFR3 has been implicated in various cancers through multiple signaling pathways. It has been reported that EXOC8 fosters oncogenic ras-mediated tumorigenesis (36). There were also some down-regulated mRNAs that were not predicted to be direct target genes of miR-99a, and we speculated that their alterations involved multiple factors and were the indirect consequences of miR-99a overexpression, as were the up-regulated mRNAs. Biological pathway enrichment analysis revealed that down-regulated mRNAs were enriched in the FGFR3 ligand binding and activation pathway and the glypican pathway, which were involved in tumorigenesis (31, 37).

It has been reported that lncRNAs and circRNAs could function as microRNA sponges and thereby regulate gene expression at a post-transcriptional level. We detected alterations in some miRNAs, lncRNAs, and circRNAs. We observed an 7.95-fold upregulation of miR-99a-5p and proved the successful construction of a stable MCF-7 cell line over-expressing miR-99a. In addition, miR-451a showed a 9.37-fold upregulation and a high expression level in our sequencing results. Over-expression of miR-451a could inhibit cell proliferation and enhance tamoxifen sensitivity in breast cancer by regulating 14-3-3ζ, estrogen receptor α, and macrophage migration inhibitory factor (38, 39). The expression of miR-210-3p was very high in both samples and showed marked down-regulation in MCF-7 cells over-expressing miR-99a compared to scramble-control cells. A global microRNA expression profile identified that miR-210 is associated with poor clinical outcomes in breast cancer patients (40) and that this molecule could interact with FBXO3 to promote breast cancer cell proliferation and migration (41). Our data indicated a 39.9-fold increase of LINC00657 in MCF-7 cells over-expressing miR-99a, which was associated with poor overall survival in human breast cancer (42). TINCR was one of the most substantially down-regulated lncRNAs. As an oncogenic lncRNA, TINCR has been identified as a subtype-specific lncRNA associated with the triple-negative and luminal B subtypes of breast cancer (43). We also detected changes in some circRNAs; however, the functions of most circRNAs are not well-characterized.

Through our cellular functional experiments, we demonstrated that miR-99a is a tumor suppressor gene. The deep sequencing results showed a few molecules thought to be oncogenes were up-regulated, while some RNAs that may act as tumor suppressors were down-regulated. However, this inconsistency does not affect our previous speculation. We constructed a ceRNA network to connect these molecules and found that lncRNAs and circRNAs harbor miRNAs and therefore indirectly regulate the expression of mRNAs. Post-transcriptional regulation makes it possible that the expression levels of oncogenes were increased or that the expression levels of anti-oncogenes were decreased after miR-99a overexpression. We conjecture that the mechanism by which miR-99a affects non-coding RNAs may be due to the regulation of transcription factors which were influenced by miR-99a. These pioneering discoveries might give us new insights into the regulatory mechanisms of microRNA.

Taken together, our findings support an important role for miR-99a in breast cancer, both as a regulator of oncogene FGFR3 and as a tumor suppressor that results in a significant decrease in breast cancer cell proliferation, invasion, and migration. We also described the transcriptome alteration caused by miR-99a, which could lay the foundation for further research concerning this molecule in breast cancer. More research is needed on miR-99a as a potential diagnostic or prognostic biomarker.

## Data Availability Statement

The datasets generated for this study are available on request to the corresponding author.

## Ethics Statement

This animal study was reviewed and approved by the Animal Ethics Committee of Wuhan Myhalic Biotechnology Co., Ltd. Written informed consent was obtained from the owners for the participation of their animals in this study.

## Author Contributions

XL, YS, and YT took part in research design. YS, PY, and XL conducted experiments and drafted the manuscript. JG, QZ, and YT participated in the data analysis and helped to draft the manuscript. XL and YT took part in research funding. All authors read and approved the final manuscript.

### Conflict of Interest

The authors declare that the research was conducted in the absence of any commercial or financial relationships that could be construed as a potential conflict of interest.

## Supplementary Material

Figure S1 Overexpression of miR-99a inhibited proliferation of MDA-MB-231 cells. **(A)** qRT-PCR of miR-99a in MDA-MB-231 cells transfected with miR-99a or the scramble control sequence. **(B)** Cell viability was determined by CCK8 assay in MDA-MB-231 cells. **(C)** Cell viability was determined by colony formation assay in MDA-MB-231 cells. Means of three independent experiments ± SEM were shown (^**^*P* < 0.01).

Figure S2 Overexpression of miR-99a inhibited migration and invasion of MDA-MB-231 cells. **(A)** Transwell migration and matrigel invasion assays on MDA-MB-231 cells transfected with miR-99a or scramble-control. **(B)** Wound healing assay on MDA-MB-231 cells in which miR-99a over-expression or not. Wounds detected in 0h and 48h were represented as a graph. Means of three independent experiments ±SEM were shown (^**^*P* < 0.01).

Figure S3 **(A)** FGFR3 silencing inhibited proliferation of MDA-MB-231 cells. MDA-MB-231 cells were transfected FGFR3 siRNA (siR-FGFR3) or the scramble control siRNA. Cell viability was determined by CCK8 assay in MDA-MB-231 cells. **(B)** FGFR3 silencing inhibited migration and invasion of MDA-MB-231 cells. Transwell migration and matrigel invasion assays on MDA-MB-231 cells were transfected FGFR3 siRNA or the scramble control siRNA. Means of three independent experiments ± SEM were shown (^**^*P* < 0.01).
