# Supplementary figures and images for: MicroRNA-99a Suppresses Breast Cancer Progression by Targeting FGFR3
Source: Front Oncol. 2020 Jan 24;9:1473. doi: 10.3389/fonc.2019.01473 (PMC6993250; doi:10.3389/fonc.2019.01473)

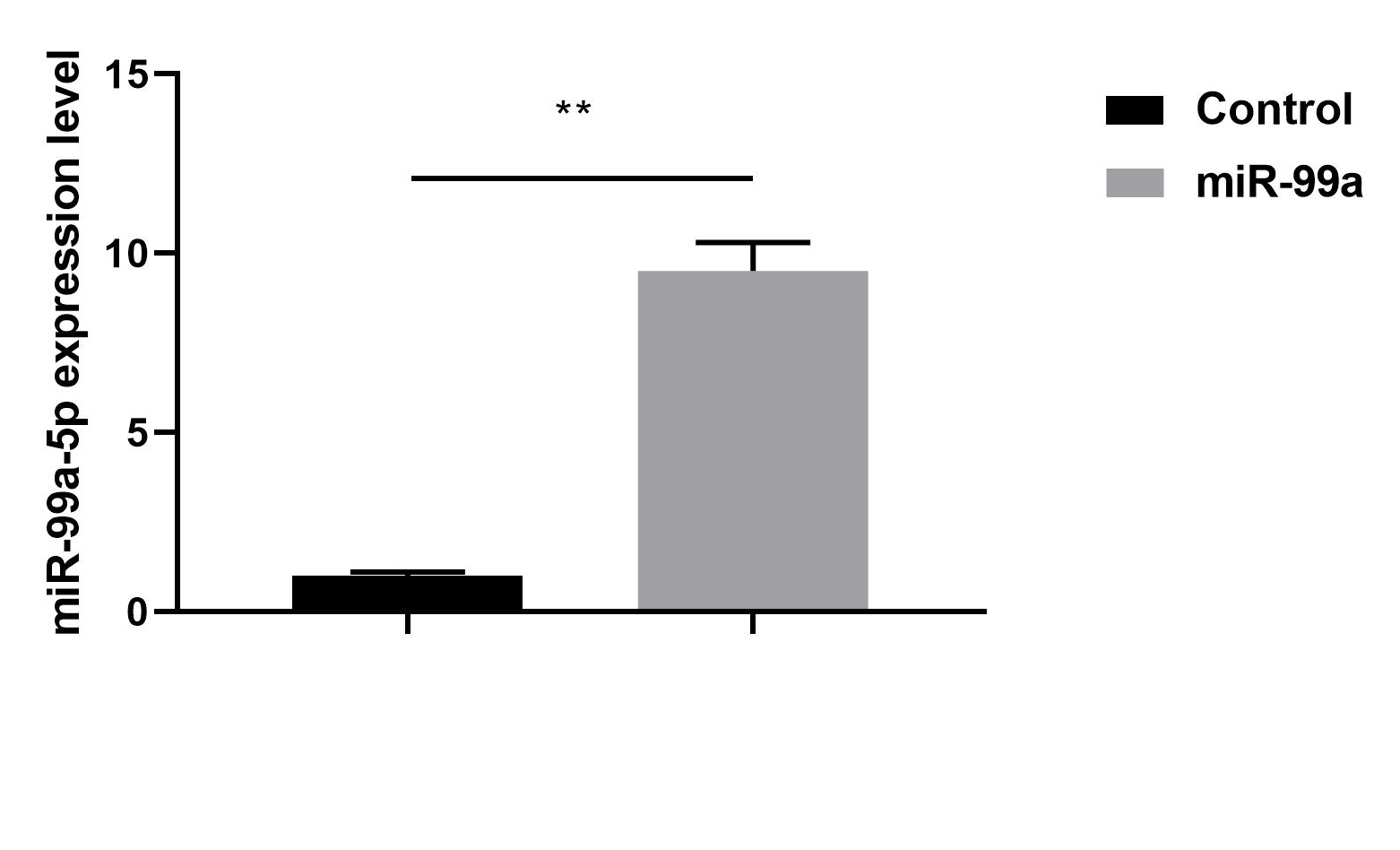

Supplement: Figure S1 — Overexpression of miR-99a inhibited proliferation of MDA-MB-231 cells. (A) qRT-PCR of miR-99a in MDA-MB-231 cells transfected with miR-99a or the scramble control sequence. (B) Cell viability was determined by CCK8 assay in MDA-MB-231 cells. (C) Cell viability was determined by colony formation assay in MDA-MB-231 cells. Means of three independent experiments ± SEM were shown (**P < 0.01). [file Data_Sheet_1.zip › Figure S1A.jpg]

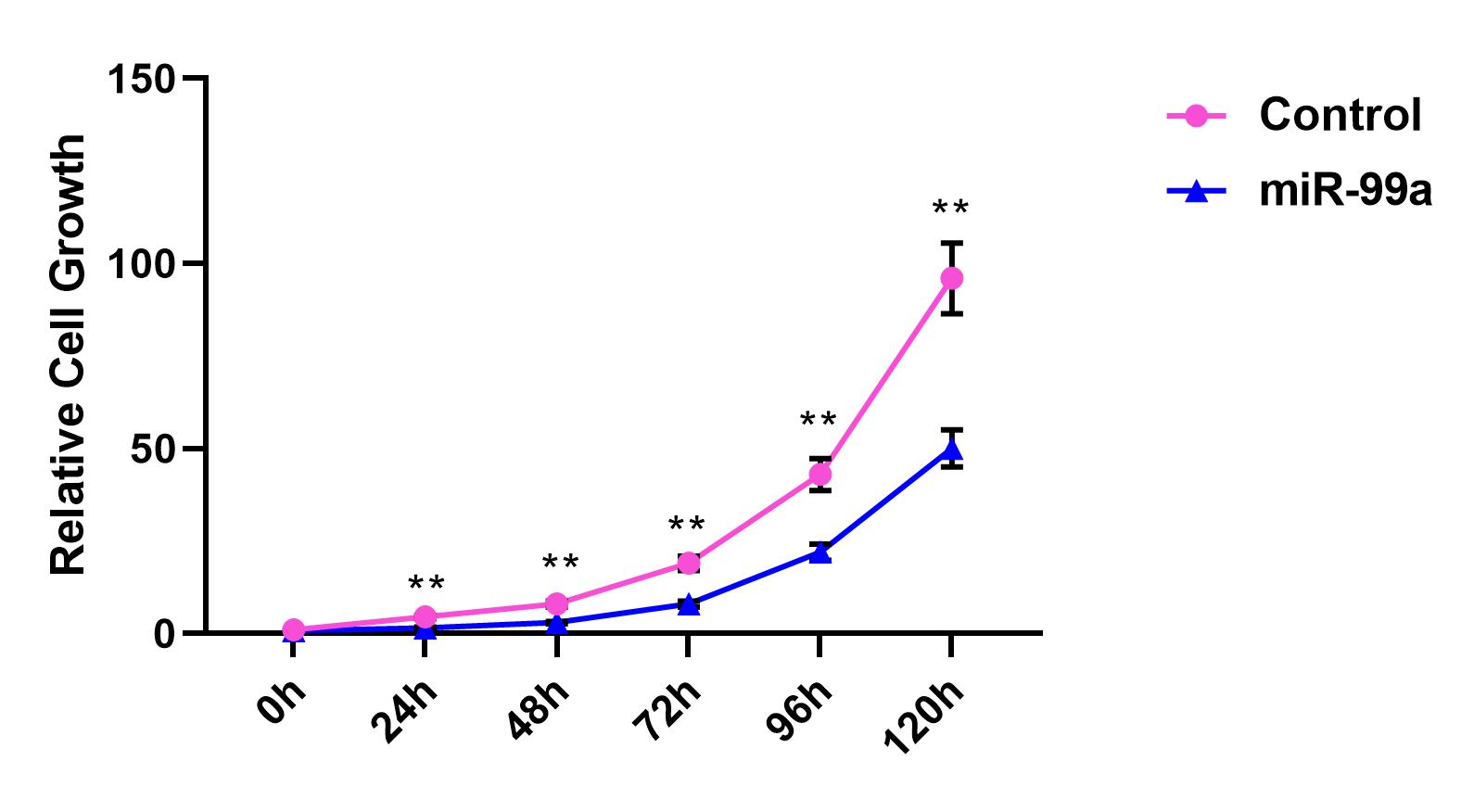

Supplement: Figure S1 — Overexpression of miR-99a inhibited proliferation of MDA-MB-231 cells. (A) qRT-PCR of miR-99a in MDA-MB-231 cells transfected with miR-99a or the scramble control sequence. (B) Cell viability was determined by CCK8 assay in MDA-MB-231 cells. (C) Cell viability was determined by colony formation assay in MDA-MB-231 cells. Means of three independent experiments ± SEM were shown (**P < 0.01). [file Data_Sheet_1.zip › Figure S1B.jpg]

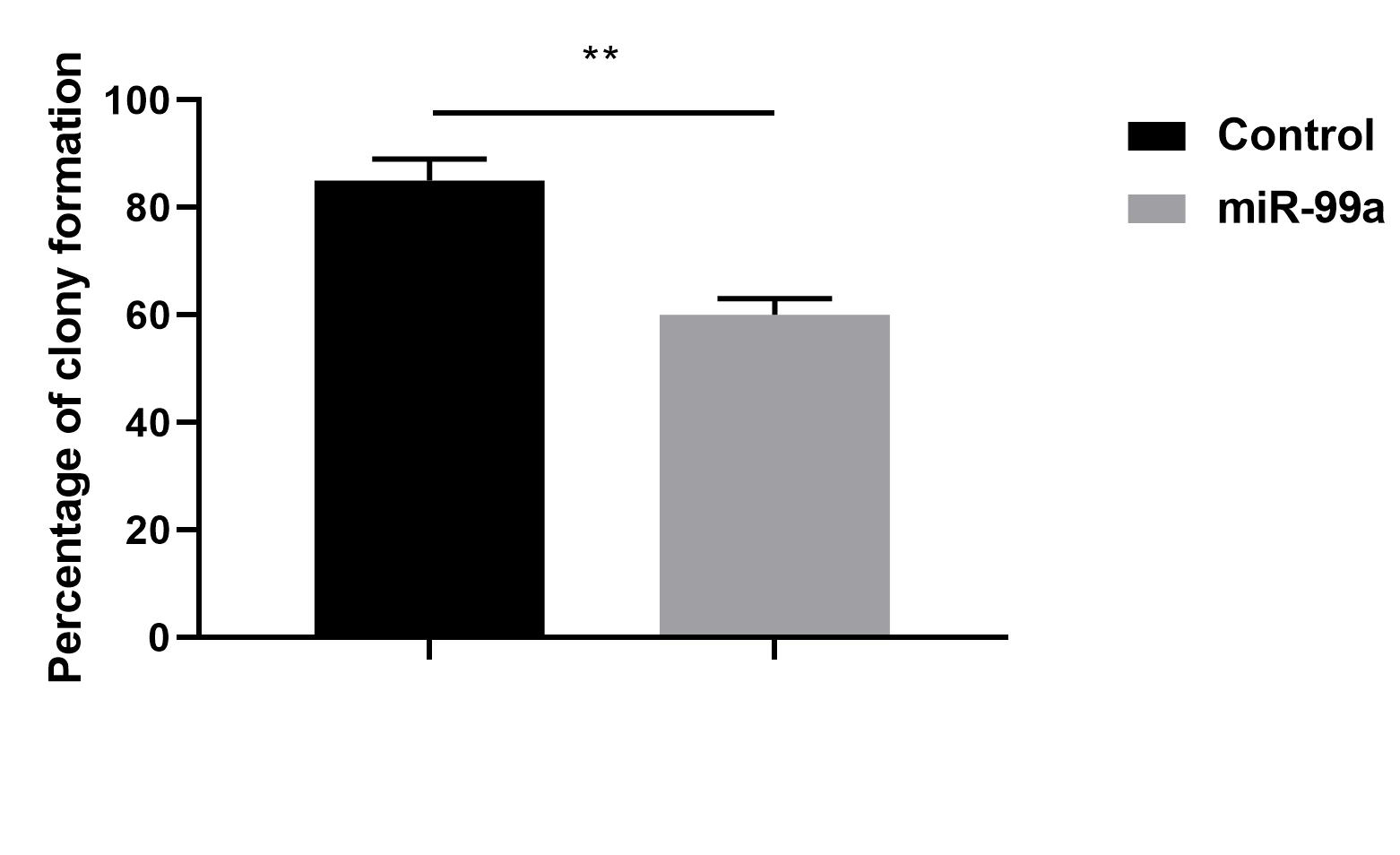

Supplement: Figure S1 — Overexpression of miR-99a inhibited proliferation of MDA-MB-231 cells. (A) qRT-PCR of miR-99a in MDA-MB-231 cells transfected with miR-99a or the scramble control sequence. (B) Cell viability was determined by CCK8 assay in MDA-MB-231 cells. (C) Cell viability was determined by colony formation assay in MDA-MB-231 cells. Means of three independent experiments ± SEM were shown (**P < 0.01). [file Data_Sheet_1.zip › Figure S1C.jpg]

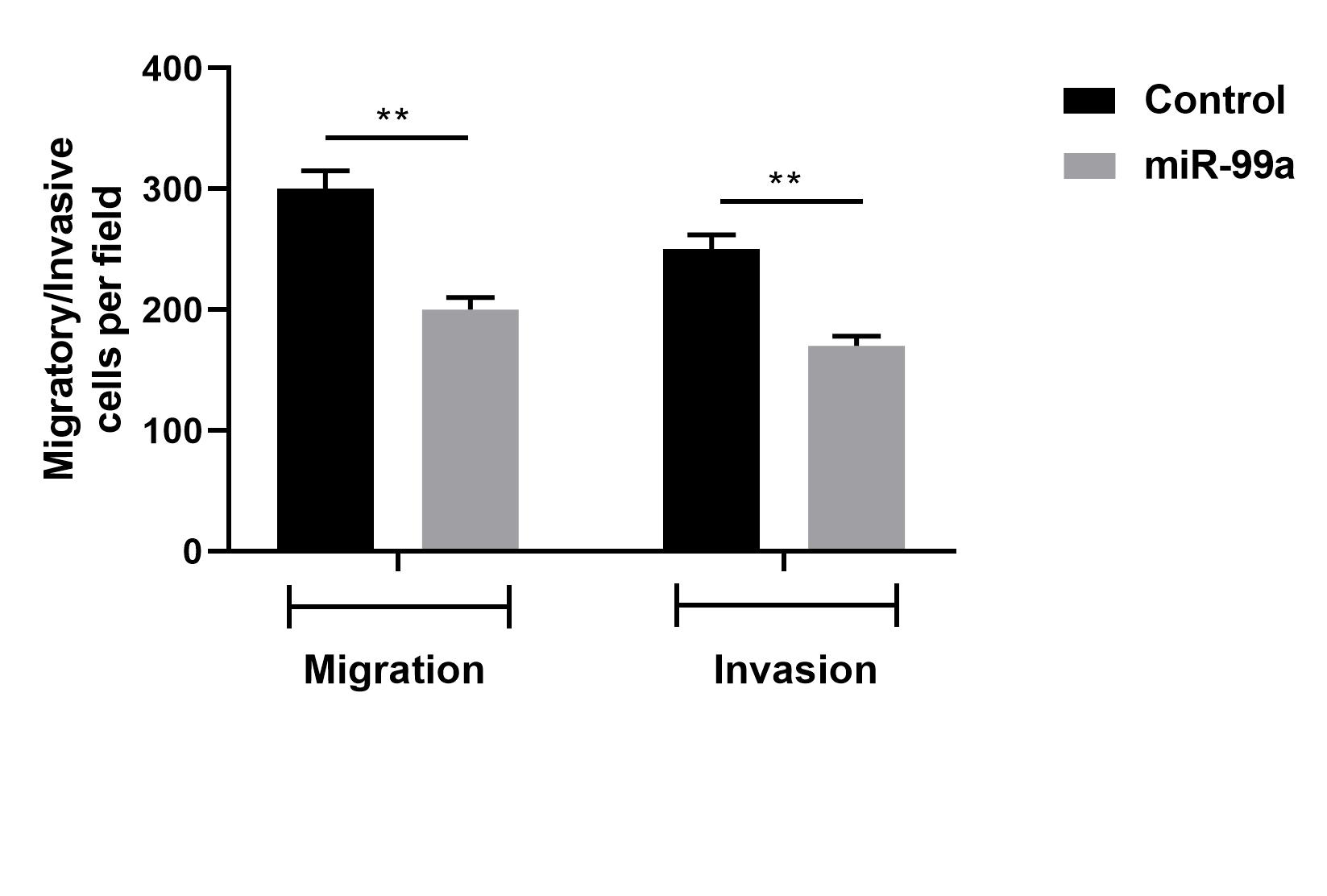

Supplement: Figure S1 — Overexpression of miR-99a inhibited proliferation of MDA-MB-231 cells. (A) qRT-PCR of miR-99a in MDA-MB-231 cells transfected with miR-99a or the scramble control sequence. (B) Cell viability was determined by CCK8 assay in MDA-MB-231 cells. (C) Cell viability was determined by colony formation assay in MDA-MB-231 cells. Means of three independent experiments ± SEM were shown (**P < 0.01). [file Data_Sheet_1.zip › Figure S2A.jpg]

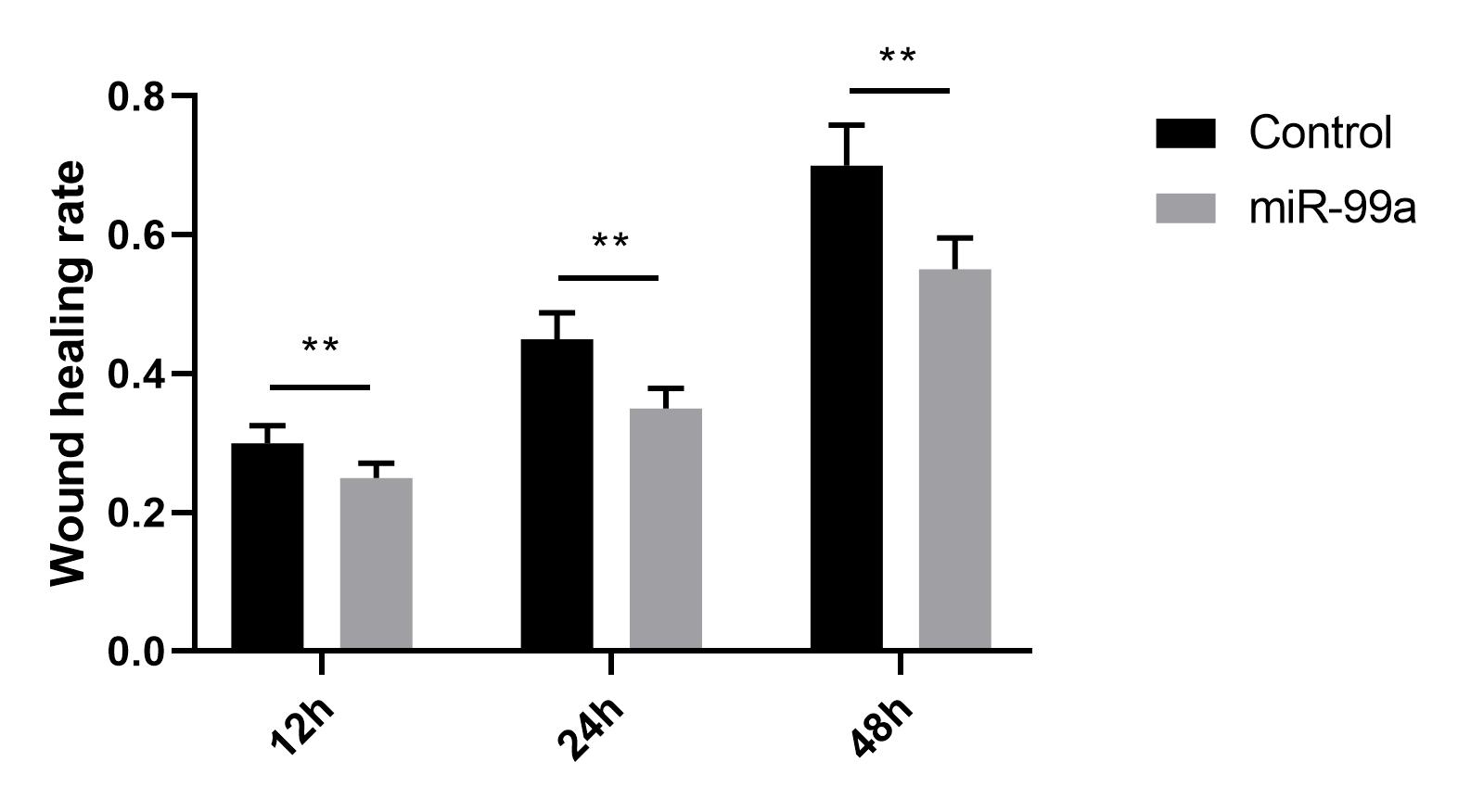

Supplement: Figure S1 — Overexpression of miR-99a inhibited proliferation of MDA-MB-231 cells. (A) qRT-PCR of miR-99a in MDA-MB-231 cells transfected with miR-99a or the scramble control sequence. (B) Cell viability was determined by CCK8 assay in MDA-MB-231 cells. (C) Cell viability was determined by colony formation assay in MDA-MB-231 cells. Means of three independent experiments ± SEM were shown (**P < 0.01). [file Data_Sheet_1.zip › Figure S2B.jpg]

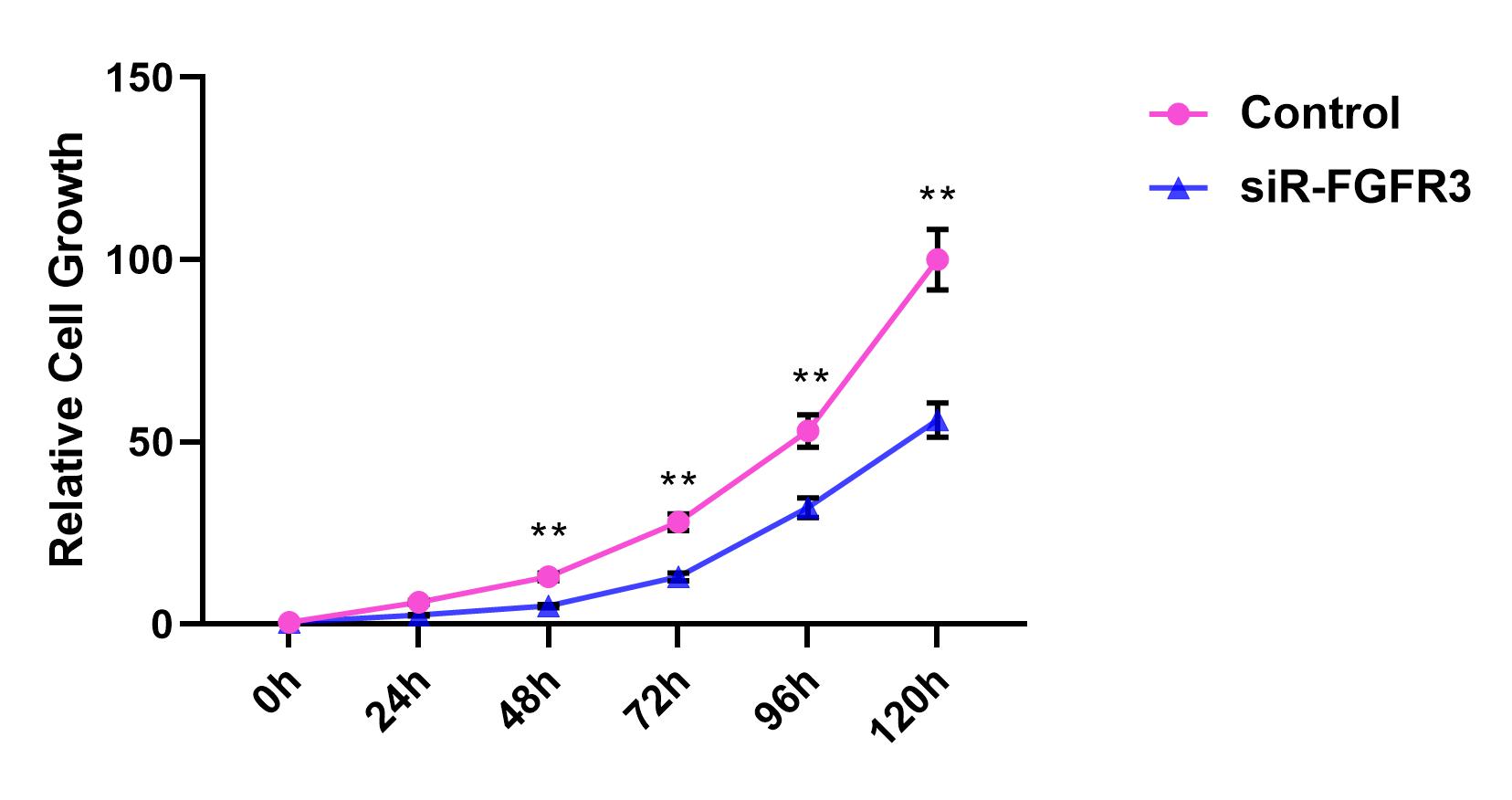

Supplement: Figure S1 — Overexpression of miR-99a inhibited proliferation of MDA-MB-231 cells. (A) qRT-PCR of miR-99a in MDA-MB-231 cells transfected with miR-99a or the scramble control sequence. (B) Cell viability was determined by CCK8 assay in MDA-MB-231 cells. (C) Cell viability was determined by colony formation assay in MDA-MB-231 cells. Means of three independent experiments ± SEM were shown (**P < 0.01). [file Data_Sheet_1.zip › Figure S3A.jpg]

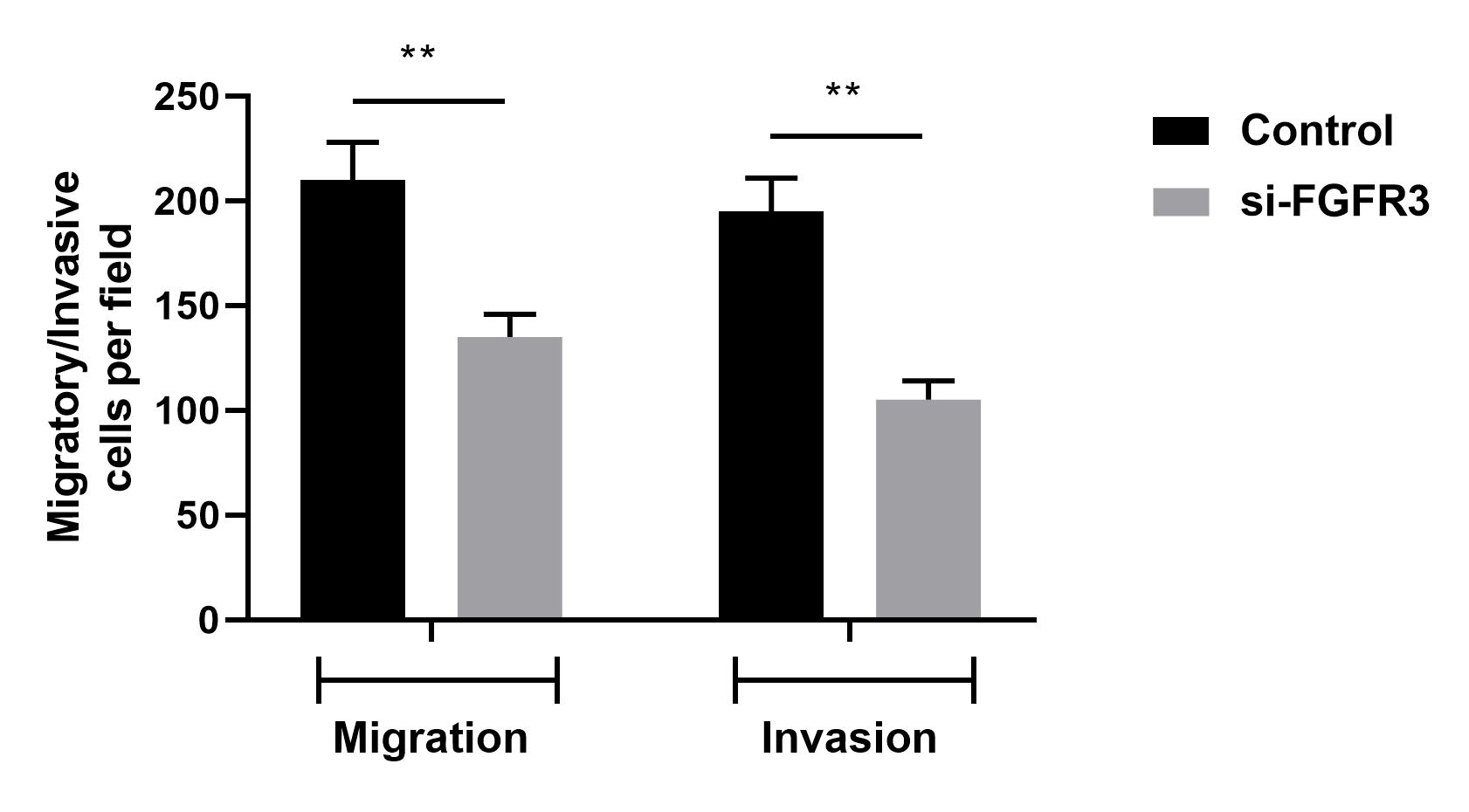

Supplement: Figure S1 — Overexpression of miR-99a inhibited proliferation of MDA-MB-231 cells. (A) qRT-PCR of miR-99a in MDA-MB-231 cells transfected with miR-99a or the scramble control sequence. (B) Cell viability was determined by CCK8 assay in MDA-MB-231 cells. (C) Cell viability was determined by colony formation assay in MDA-MB-231 cells. Means of three independent experiments ± SEM were shown (**P < 0.01). [file Data_Sheet_1.zip › Figure S3B.jpg]
